# Decreased expression of extracellular matrix proteins and trophic factors in the amygdala complex of depressed mice after chronic immobilization stress

**DOI:** 10.1186/1471-2202-13-58

**Published:** 2012-06-06

**Authors:** Soonwoong Jung, Younghyurk Lee, Gyeongwha Kim, Hyeonwi Son, Dong Hoon Lee, Gu Seob Roh, Sang Soo Kang, Gyeong Jae Cho, Wan Sung Choi, Hyun Joon Kim

**Affiliations:** 1 Department of Anatomy and Neurobiology, Institute of Health Sciences, Medical Research Center for Neural Dysfunction School of Medice, Gyeongsang National University, 92 Chilam-dong, Jinju, 660-751, South Korea

## Abstract

**Background:**

The amygdala plays an essential role in controlling emotional behaviors and has numerous connections to other brain regions. The functional role of the amygdala has been highlighted by various studies of stress-induced behavioral changes. Here we investigated gene expression changes in the amygdala in the chronic immobilization stress (CIS)-induced depression model.

**Results:**

Eight genes were decreased in the amygdala of CIS mice, including genes for neurotrophic factors and extracellular matrix proteins. Among these, osteoglycin, fibromodulin, insulin-like growth factor 2 (Igf2), and insulin-like growth factor binding protein 2 (Igfbp2) were further analyzed for histological expression changes. The expression of osteoglycin and fibromodulin simultaneously decreased in the medial, basolateral, and central amygdala regions. However, Igf2 and Igfbp2 decreased specifically in the central nucleus of the amygdala. Interestingly, this decrease was found only in the amygdala of mice showing higher immobility, but not in mice displaying lower immobility, although the CIS regimen was the same for both groups.

**Conclusions:**

These results suggest that the responsiveness of the amygdala may play a role in the sensitivity of CIS-induced behavioral changes in mice.

## Background

Stress is believed to cause various diseases, yet has also been shown to have some beneficial effects [1]. Stress can lead to somatic abnormalities and can evoke psychiatric illnesses such as anxiety disorders and major depressive disorder [2]. Therefore, to elucidate a pathway to recovery, much research has focused on investigating why various stresses result in such diseases [3].

Recent clinical and pre-clinical studies have revealed that psychiatric illnesses such as depression and anxiety are due to over-activated and/or depressed functions of specific brain regions. Particular emphasis has been placed on examining the prefrontal cortex, hippocampus, and amygdala due to the different structure and function of these regions in psychiatric cases compared with normal controls [4,5]. Notably, the importance of the amygdala in this pathway has been emphasized because of its many neural connections to other brain regions [6].

The amygdala was named for its almond-like shape and is known for its role in the formation and recall of emotional memories [7]. Because the amygdala consists of four nuclei (lateral, medial, central, and basal nucleus), it is also often referred to as the amygdala complex. Each nucleus has its own neural circuit that is distinct from the other nuclei in the amygdala complex and other brain areas. Through these neural circuits, the amygdala plays an important role in integrating and responding to external sensory information [6].

Determining the changes in the brain that occur with different mental illnesses will provide invaluable clues for understanding and formulating an earlier prognosis and developing proper treatments for anxiety disorders and depression [8]. These changes may be epigenetically evoked by environmental changes such as stress. Therefore, much work has been performed in various laboratories worldwide focusing on the elucidation of gene expression patterns and changes in experimental animal models [5,9]. Our laboratory previously established a chronic immobilization stress (CIS)-induced anxiety and depression mouse model, and changes in gene expression were reported in the prefrontal cortex [10]. In our current study, we found that a few extracellular matrix (ECM) proteins and trophic factors were reduced in the amygdala of CIS-induced depressed mice compared with control mice.

## Methods

### Analysis of amygdala gene expression in anxious and depressed mice

To analyze the gene expression pattern in the amygdala of anxious and depressed mice, we performed cDNA microarray analysis on micro-punched amygdala tissue from ICR mice experiencing CIS [10]. Briefly, 7-week-old male ICR mice were subjected to a 15-day (2 h/day) CIS paradigm, and CIS-induced behavioral changes were measured in an open field test, an elevated plus maze, and a forced swim test (FST). For microarray analysis, total RNA from the amygdala of control (CTL) and stressed (STR) mice was obtained using a micro-punch (diameter 1.21 mm, Stoelting, IL, USA) and pooled into one sample consisting of 10 mice from each group. A DNA microarray assay was done once at the DNA Microarray Core Facility (University of Kentucky, Lexington, KY) using Affymetrix equipment, protocols, and the GeneChip® Mouse Expression Set 430 2.0. CIS-responsive genes were selected if their expression values, which were signal intensities of probe hybridization provided by Affymetrix microarray assay equipment [11], changed by 2-fold or greater.

### Confirmation of changes in gene expression in the amygdala

#### Animals and treatments

To confirm gene expression changes, male C57BL/6 mice were used. Male 7-week-old C57BL/6 mice (Samtako, Co. Ltd., Korea) were habituated for 1 week before experimentation in a specific pathogen-free-grade animal facility at Gyeongsang National University School of Medicine. Temperature and humidity were 22 °C and 50–60%, respectively, and mice were reared under a 12-h light/dark cycle (lights on at 6 a.m.). Mice were supplied with free access to laboratory chow and water. CIS was carried out as previously described [10]. Briefly, mice were repeatedly placed in a restrainer for 2 h/day for 15 days under 200 lux light conditions. Body weight and food intake were evaluated every other day. All procedures were performed in accordance with an approved protocol (GLA-100917-M0093) by Gyeongsang National University Institution Animal Care & Use Committee (GNU IACUC).

### Behavioral assessments

**Figure 1:**
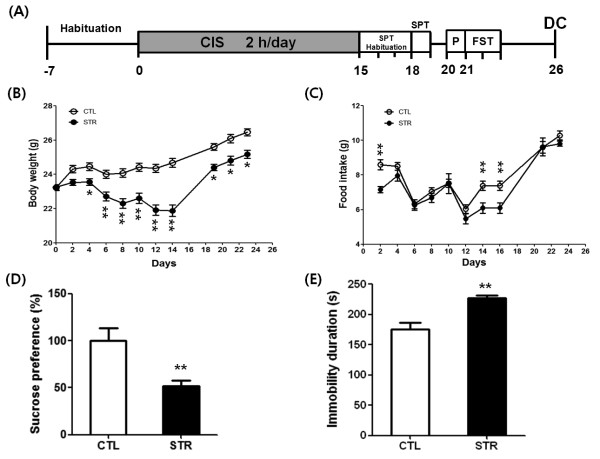
**A schematic CIS paradigm and the result of behavioral assessments. (A)** A schematic drawing of CIS (2 h per day for 15 days) and behavioral assessments: sucrose preference test (SPT) and forced swim test (FST). After behavioral assessments, the experimental animals were killed by decapitation (DC) on day 26 for Q-PCR analysis. On the day before FST, mice were adapted to swimming with a pre-swimming (P) session. The body weight **(B)** and food intake **(C)** were measured every other day during experimentation except for during the SPT period. The significance of the mean difference between the stressed (STR, n = 10) and control (CTL, n = 10) groups was measured with a *t*-test on each day. The STR group showed body weight reduction over the 15-day CIS period. Significant mean differences in food intake were only found on days 2, 14, and 16 after the experiment began. Sucrose preference of the STR group was significantly decreased **(D)**, and immobility duration of the STR group increased **(E)** compared with CTL, indicating that STR mice were more depressed than CTL mice. Data are the means ± SEM. *, P < 0.05; **, P < 0.01 between CTL and STR groups

To measure the depression level of CIS mice, a sucrose preference test (SPT) and FST were conducted as depicted in Figure 1A. The SPT was performed with some modifications as previously described [12] to determine symptoms of anhedonia. Briefly, mice were habituated for 48 h with 0.1 M sucrose solution. Following a 12-h water deprivation period, consumption of sucrose solution and water was determined for 6 h. The sucrose preference was represented as the ratio of sucrose-to-water consumption. The FST method was performed as previously described [10]. Mice were subjected to pre-swimming for 5 min on the day prior to experimentation. Mobility was defined as a change in 5% of the recorded pixels (EthoVision, Noldus Information Technology, The Netherlands). The water in the chamber was changed between each mouse.

### Quantitative PCR analysis

Mice were anesthetized with CO_2_ (CTL; n = 9, STR; n = 9) and decapitated. Brains were removed and quickly frozen in isopentane pre-chilled in liquid nitrogen. Amygdaloid tissue was obtained using a micro-punch 0.8 mm deep from the caudal end forward to −1.58 mm from the bregma [13]. Tissue punch samples were stored at −70 °C until use. Total RNA was extracted using Trizol reagent and further purified using an RNeasy kit (Qiagen Inc., Valencia, CA, USA). To verify the microarray results, quantitative PCR (Q-PCR) was performed with gene-specific primers (Table 1). First strand cDNA was synthesized from 1 μg total RNA with oligo (dT)_15_ primers. Q-PCR amplification and relative quantification were achieved using a LightCycler 480 (Roche) as previously described [14]. The data are presented as mRNA expression levels and were normalized to GAPDH expression as an internal control.

**Table 1:** Primers used for Q-PCR analysis

**Gene Name**	**Primer sequence (5′- 3′)**		**Reference or GenBank Accession number**
*Fibromodulin*	Forward	GAG CCT CTG CTC ATC CTT TG	[15]
	Reverse	CTG GTT TGG CTT TTG TGG AT	
*Osteoglycin*	Forward	TGA TGC TGT ACC ACC ATT GC	[16]
	Reverse	ATT CCA GGT CGT TAT GGT CC	
*Procollagen C-proteinase enhancer protein*	Forward	CTG AGC ACC AGT TTT GTG GG	NM 008788
	Reverse	CCA GCC TCT TTG AGT CGT CA	
*Procollagen, type I, alpha 2*	Forward	CTG CTG GAG TCA AGG GTG AT	NM 007743
	Reverse	CTG TCT CCT TGC TTG CCA GT	
*Thrombomodulin*	Forward	ATG CGT GGA GCA TGA GTG	[17]
	Reverse	CTG GCA TCG AGG AAG GTC	
*Growth differentiation factor 10*	Forward	GGT GGA CTT CGC AGA CAT CG	NM 173404
	Reverse	GAT GGT GGC ATG GTT GGA TG	
*Insulin-like growth factor 2*	Forward	CGC TTC AGT TTG TCT GTT CGG	[18]
	Reverse	TGG GTG GTA ACA CGA TCA GG	
*Insulin-like growth factor binding protein 2*	Forward	GGC GCG GGT ACC TGT GAA AA	[18]
	Reverse	TCT CCT GCT GCT CGT TGT AG	
*Insulin-like growth factor binding protein 4*	Forward	CCA TCC AGG AAA GCC TGC AG	[18]
	Reverse	TGG AAG TTG CCG TTG CGG TCA CAG	
*Gapdh*	Forward	TGC CGC CTG GAG AAA CCTG C	[14]
	Reverse	TGA GAG CAA TGC CAG CCC CA	

### Immunohistochemistry

Immunohistochemistry was performed as previously described [19], with several modifications. Briefly, fixed brains were sectioned at 30-μm thickness and incubated with anti-fibromodulin (Santa Cruz Biotechnology, Cat #; L2807, 1:100), osteoglycin (Santa Cruz Biotechnology, Cat #; A0108, 1:100), insulin-like growth factor 2 (Igf2; Abcam, Cat #; ab63984, 1:200), or insulin-like growth factor binding protein 2 (Igfbp2; R&D Systems, Cat #; AF797, 1:100) at 4 °C overnight. Signals were visualized using Alexa Flour-conjugated secondary antibodies, and nuclei were stained with DAPI. Signal specificity was confirmed with a negative control; sections that were incubated with no primary antibody showed no specific signals. Digital images were captured and documented (Olympus, Tokyo, Japan). The signal densities were analyzed on similar sections from three different mice from each group with an image processing program (NIH Image J, Bethesda, MD).

### Statistics

Data were evaluated using a one-way ANOVA and a *post-hoc* (Tukey’s multiple-comparison test) test (GraphPad Prism, Ver 5.01). The *t*-test was used to compare two groups (SigmaStat Ver. 10.0). Statistical significance was set at P < 0.05.

## Results

Previously, we used microarray analysis and reported cortical gene expression changes in anxious and depressed mice experiencing CIS [10]. We saved amygdala tissues from the mice in that study and performed DNA microarray analyses using them. Therefore, the microarray results presented in this study are from the amygdala of CIS-induced anxious and depressed mice.

To select genes for further study from the microarray data, we used two criteria. The first was a gene expression change of more than 2-fold, and the second was an expression value greater than 500 in either group of control or stressed animals. The expression value was hybridization signal intensity provided by the Affymetrix microarray system software. Consequently, only 10 probes (eight genes) satisfied the criteria (Table 2), a number that was very small compared to the large number of probes on the chip. Also, these genes were functionally categorized into only two groups, ECM and trophic factors. Fibromodulin, osteoglycin, thrombomodulin, procollagen C-proteinase enhancer protein, and procollagen type 1, alpha 2 are ECM proteins; growth differentiation factor 10 (Gdf10), Igfbp2, and Igfbp4 belong to the trophic factor family. Because the microarray was done once without replicate, confirmation of gene expression changes should be done with further analyses.

**Table 2:** List of known genes that showed a 2-fold or greater change in expression in the amygdala of CIS-induced depressed mice

**GenBank Accession No.**	**Gene Description**	**Expression Values**	
		**Control**	**Stress**
NM_021355.1	fibromodulin	***603***	***186***
BB532202	fibromodulin	***1275***	***523***
BC021939.1	osteoglycin	***737***	***249***
BB542051	osteoglycin	***705***	***252***
BF227507	procollagen, type 1, alpha 2	***563***	***203***
BB250811	procollagen C-proteinase enhancer protein	***508***	***248***
NM_009378.1	thrombomodulin	***507***	***247***
L42114.1	growth differentiation factor 10	***565***	***279***
AK011784.1	insulin-like growth factor binding protein 2	***1158***	***544***
BB787243	insulin-like growth factor binding protein 4	***580***	***236***

To confirm the gene expression changes of selected genes using another experimental animals and Q-PCR analyses, we produced CIS-induced depressed mice using a similar yet slightly different paradigm (Figure 1A) from our previous study [10]. We wanted to focus only on the depression-like behaviors evoked by CIS. For this, two behavioral tests, SPT and FST, were conducted to assess the depression level of the mice after a 15-day CIS treatment. In addition, we replaced the ICR mouse strain with C57BL/6, because the latter are more popularly used. During the CIS procedure, body weight and food intake were checked every other day (Figure 1B and C). We observed a significant reduction in body weight in the stressed group compared with controls, although there was no significant difference in their food intake during the mid-stage of CIS treatment. Therefore, the body weight change may have been due to differences in energy expenditure between the control and stressed groups. As expected, the stressed group showed a lower sucrose preference and higher immobility duration than the control group, implying increased depression in the stressed group (Figure 1D and E).

**Figure 2:**
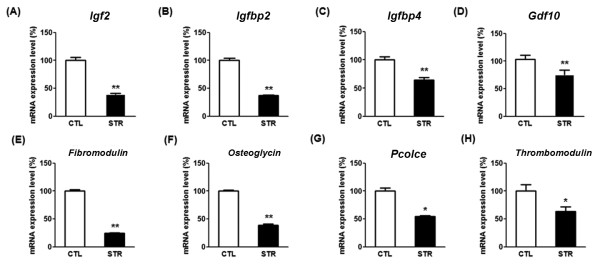
**The results of Q-PCR analysis of selected genes using total RNA from the amygdala from stressed (STR, n = 9) and control (CTL, n = 9) mice.** Insulin-like growth factor 2 (*Igf2*) **(A)**, Insulin-like growth factor binding protein-2 (*Igfbp2*) **(B)**, *Igfbp4***(C)**, growth differentiation factor 10 (*Gdf10*) **(D)**, fibromodulin **(E)**, osteoglycin **(F)**, procollagen C-proteinase enhancer protein (*Pcolce*) **(G)**, and thrombomodulin **(H)** mRNAs were significantly decreased in the amygdala of STR mice. Data are the means ± SEM. *, P < 0.05; **, P < 0.01 between the CTL and STR groups

After confirmation of behavioral changes, Q-PCR was conducted to verify mRNA expression changes in the CIS-induced depressed animals. Since the list (Table 2) contained two genes of Igf-binding proteins, *Igfbp2* and *Igfbp4*, we hypothesized that the expression of *Igf*-related genes could be influenced by CIS. We again analyzed the microarray data to see whether other *Igf*-related genes were changed. It was found that *Igf2* expression was decreased by 1.7-fold with high expression level (expression value of control group was 1449.4) and other *Igf*-related genes showed neither remarkable changes nor high expression level (data not shown). Therefore, we included *Igf2* in Q-PCR analysis. Because the change in expression was less than 2-fold, the genes listed in Table 2 do not include *Igf2*. From Q-PCR analysis, however, *Igf2* showed significantly lower expression in the STR group compared with the CTL, which was similar to other gene candidates (Figure. 2). However, mRNA expression of procollagen type I, alpha 2, a gene in Table 2, did not significantly differ between the STR and CTL groups (data not shown). As a result, seven genes from Table 2 and *Igf2* were decreased in the amygdala of CIS-induced depressed mice, and thus, we identified a total of eight genes as responders to CIS in the mouse amygdala. The microarray results obtained from ICR mice were confirmed in C57BL/6 mice with Q-PCR, indicating that the CIS-induced gene expression change in the amygdala did not differ between the two mouse strains.

**Figure 3:**
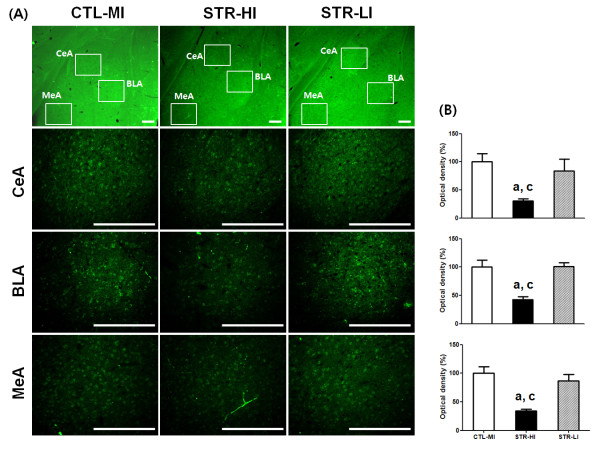
**The expression of osteoglycin in the amygdala was analyzed using immunohistochemistry (IHC) in the three groups; CTL-MI, STR-HI, and STR-LI**. The representative IHC result shows specific decreased expression of osteoglycin in the central (CeA), basolateral (BLA), and medial (MeA) nuclei of the amygdala of STR-HI mice **(A).** The fluorescence signal density of each nucleus was quantified using the Image J program (NIH), **(B)** and the strength of IHC was evaluated with one-way ANOVA and Tukey’s multiple-comparison test among groups. All tested nuclei showed significant differences in signal intensity among groups (F values of CeA, BLA, and MeA were 8.79, 14.70, and 13.55, respectively). As a result, only STR-HI mice showed significant signal decreases in each nucleus. Data are the means ± SEM (n = 3). a, P < 0.05 between CTL-MI and STR-HI mice; c, P < 0.05 between STR-HI and STR-LI mice. Scale bars are 200 μm

**Figure 4:**
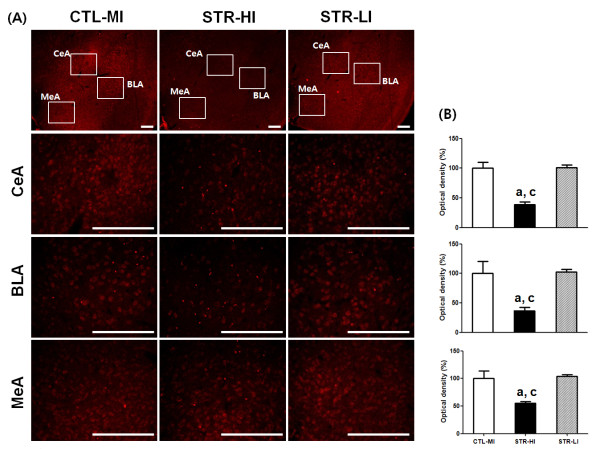
**The expression of fibromodulin in the amygdala was analyzed using IHC in the three groups; CTL-MI, STR-HI, and STR-LI mice.** The results of IHC showed specific decreased expression of fibromodulin in the CeA, BLA, and MeA nuclei of the amygdala in STR-HI mice **(A)**. The fluorescence signal density of each nucleus was quantified using the Image J program (NIH), **(B)** and the strength of IHC was evaluated with one-way ANOVA and Tukey’s multiple comparison test among groups. All tested nuclei showed significant differences of signal intensity among groups (F values of CeA, BLA, and MeA are 26.07, 8.915, and 10.98, respectively). As a result, only the STR-HI group showed significant signal decreases in each nucleus. Data are the means ± SEM (n = 3). a, P < 0.05 between CTL-MI and STR-HI; c, P < 0.05 between STR-HI and STR-LI mice. Scale bars are 200 μm

**Figure 5:**
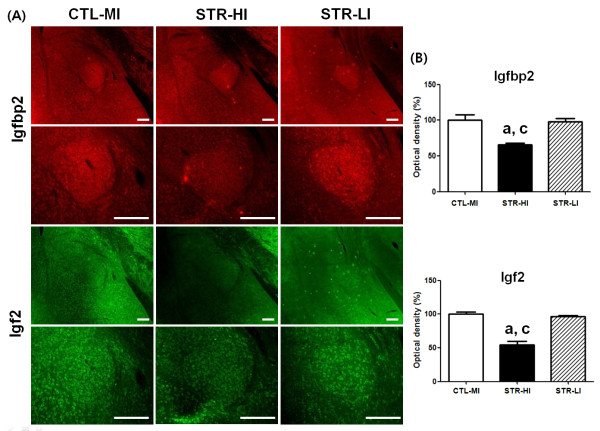
**The expression of Igfbp2 and Igf2 in the amygdala was analyzed using IHC in the three groups; CTL-MI, STR-HI, and STR-LI mice.** IHC showed specific decreased expression of Igfbp2 and Igf2 in the CeA nucleus of STR-HI mice **(A)**. The fluorescence signal density was quantified using the NIH Image J program, and the strength of IHC was evaluated with one-way ANOVA and Tukey’s multiple comparison tests among groups. Igfbp2 (F = 13.52) and Igf2 (F = 53.25) showed statistically significant differences among groups with one-way ANOVA. Tukey’s post tests revealed that only the STR-HI mice had a significant decrease in two proteins in the CeA **(B)**. Data are the means ± SEM (n = 3). a, P < 0.05 between CTL-MI and STR-HI mice; c, P < 0.05 between STR-HI and STR-LI mice. Scale bars are 200 μm

In our laboratory, CIS-induced depressed mice displayed a somewhat wide range of behavior, although the mean value of immobility of the STR group was statistically different from the CTL group. For example, a few mice showed a relatively lower duration of immobility, which was comparable to the duration of the higher immobility CTL group. We used to exclude these lower duration mice from the CIS-induced depressed group for further experimentation. In the present study, for histological examination, we prepared new model animals according to the scheme depicted in Figure 1A and confirmed significant mean differences both in sucrose preference and immobility duration between the CTL and STR groups (Table 3). Then we selected six STR mice from two end points of the immobility spectrum and three mice from the CTL group to see whether CIS-responsive gene expression was different according to the behavioral observations. Three mice showing the highest immobility duration from the STR group were categorized as STR-higher immobility (HI) mice, and three mice showing the lowest immobility duration from the STR group were considered STR-lower immobility (LI) mice. CTL-mean immobility (MI) mice consisted of mice with immobility duration in the mean range of the CTL group. From immunohistochemistry (IHC), we found that the overall expression level of osteoglycin was decreased in the amygdala of the STR-HI mice, specifically in the central, basal, and medial parts compared with the CTL-MI mice (Figure 3). However, there was no significant difference between the CTL-MI and STR-LI mice. The expression of fibromodulin was significantly decreased in these three parts of the amygdala in STR-HI mice but not in the STR-LI mice, and this pattern was very similar to that of osteoglycin (Figure 4). Protein expression of Igf2 and Igfbp2 appeared to be specific to the central nucleus in the amygdala, and expression was also decreased only in the STR-HI mice (Figure 5).

**Table 3:** The effect of chronic immobilization stress on mouse depression-like behaviors. (Results are expressed as mean ± SEM)

**Behavioral Parameters**	**Control**	**Stress**	***P* value**
	**(n = 10)**	**(n = 15)**	
Sucrose Preference (%)	104.7 ± 4.9	79.4 ± 5.8	<0.001
(Sucrose Preference Test)			
Range	80-130	54-105	
Immobility Duration (s)	137 ± 5.3	178.6 ± 6.5	<0.001
(Forced Swim Test)			
Range	111-159	125-209	

**Figure 6:**
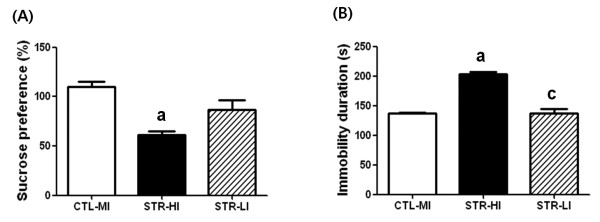
**The sucrose preference and immobility duration of the mice selected for immunohistochemical analyses.** Statistical significance was evaluated with one-way ANOVA and Tukey’s *post-hoc* test. There were significant differences in sucrose preference (**A**, F = 13.30, P = 0.0062) and immobility duration (**B**, F = 52.59, P = 0.0002) among the groups. From Tukey’s *post-hoc* tests, STR-HI mice showed a statistically significant difference compared to the CTL-MI mice in the two parameters but STR-LI mice did not show a significant difference from CTL-MI mice. Data are the means ± SEM (n = 3 per group). a, P < 0.05 between CTL-MI and STR-HI mice; c, P < 0.05 between STR-HI and STR-LI mice

To observe the behavioral differences in mice used for IHC, we reanalyzed these data as shown in Figure 6. STR-HI mice showed a significant difference in immobility duration compared to CTL-MI and STR-LI mice, and the mean value of STR-LI mice was similar to that of CTL-MI mice (Figure 6B), because this selection was done using their immobility duration data. The mean value of sucrose preference (Figure 6A) in STR-LI mice was slightly higher than STR-HI mice and lower than CTL-MI mice but the difference was not significant, indicating that mice undergoing CIS were different from those in the CTL group. However, STR-HI mice showed a significantly lower sucrose preference compared with CTL-MI mice (Figure 6A).

Statistically significant decreases in protein expression of four candidates were only found in the amygdala of STR-HI mice but not in STR-LI mice. These protein expression patterns suggest that the decreases in the amygdala may be closely correlated with the degree of behavioral changes caused by CIS.

## Discussion

Using cDNA microarray and Q-PCR analysis, we identified eight genes that were decreased in the amygdala of CIS-induced depressed mice. Four genes were further characterized using an immunohistological approach, which showed that protein expression was specifically reduced in the amygdala of STR-HI mice but not in STR-LI mice, even though both groups experienced the same CIS protocol.

Almost all organisms feel threats of homeostatic imbalance, and intrinsic stress responses begin to work during threats to cope with the stressors [20]. The stress response starts when the brain receives information about stressors through sensory mechanisms scattered throughout the body and activates specific pathways that were imprinted early in physiological development. In this way, organisms can maintain homeostatic conditions in stressful environments [6]. However, repetitive, long-lasting stress can evoke depressive behaviors directed by the brain [21]. The brain has structural and functional plasticity that can result in both adaptive and maladaptive consequences [22]. It is now widely accepted that intense emotional events or chronic exposure to stressful experiences can create traumatic memories and even result in the development of mood and anxiety disorders, including post-traumatic stress disorder (PTSD) and major depressive disorder [22-24]. Depression has been reported to be the disease for which drugs are most frequently prescribed and is notorious for leading to many disabilities [25].

Research groups that consider stress as the main cause of depression have attempted to elucidate how stress results in structural and functional changes in specific brain regions and to develop innovative prevention and therapeutic strategies based on their findings [26]. Recently, large-scale gene expression analysis has been performed using animal models of endogenous depression and chronic stress to test the monoamine hypothesis and find a common pathological mechanism [5]. However, the authors found no significant difference in the expression of monoaminergic transmission-related genes in either model, and very few overlapping, expression-changing genes in the two models, implicating divergent mechanisms between endogenous depression and stress-induced behavioral changes [5]. Although the authors failed to find a common pathological event, a few genes identified in that study as stress responsive were also found in our studies. For example, *Igf2* was identified in this study (Figure 2), and *transthyretin* was previously identified [10]. Thus, these genes could be classified into chronic stress-responsive genes, and we may be able to obtain more plausible information about how stress evokes depression by studying the roles of such genes.

We carried out a microarray assay using an Affymetrix DNA chip, the GeneChip® Mouse Expression Set 430 2.0, which has almost 40,000 probes. However, as shown in Table 2, only 10 probes were selected as CIS responders, with the criteria that expression was relatively high and more than 2-fold different between the CTL and STR groups. All probes listed in Table 2 decreased in the stressed amygdala and were categorized as ECM proteins or trophic factors by their known functions. This result was very impressive and interesting, and the nature of the amygdala suggests a possible reason. The amygdala mainly consists of four nuclei that cross-talk among each other and have neural connections with other brain areas [6]. The prefrontal cortex plays a major role in recognizing environments and making decisions, but the amygdala is a key player that stores and consolidates emotional memory and behavioral patterns [24]. Almost all external stimuli afferent to the cerebral cortex affect the amygdala complex before being processed in the cerebral cortex [6]. Therefore, the amygdala has numerous neural circuits with various brain regions and plays an important role in the process of emotional performance and memory formation [27]. In addition, ECM proteins in the brain not only have supportive and trophic functions, but modulate cell functionality and plasticity as well as participate in brain architecture [28]. Proteoglycans and collagens are important components of the brain ECM that have multiple functions, including cell adhesion, neurite outgrowth, ECM assembly, and tumor cell invasion [29]. Fibromodulin and osteoglycin are small, leucine-rich proteoglycans [30-32]. Moreover, trophic factors are generally regarded as major components that impact neural plasticity [33]. Thus, decreased expression of such genes in the amygdala may affect amygdaloid neural plasticity, including neuronal morphological changes and afferent and/or efferent neural circuits participating in stress-related emotional behaviors, which may result in psychiatric illness.

Several lines of evidence support the concept that stress-induced functional and morphological alterations in the amygdala can evoke psychiatric illness, including anxiety disorder, depressive disorder, and PTSD [22,23,34-38]. Neurons in the medial amygdala (MeA) have decreased numbers of dendritic spines after stress exposure, but neurons in the basolateral amygdala (BLA) have increased numbers [35]. The dendritic hypertrophy of the BLA induced by stress is considered a quantifiable marker of neuronal structural pathology in bipolar disorder [36]. The decrease in dendritic spines in the MeA may be a maladaptive phenotype in stress-induced psychiatric patients, including non-regulated energy homeostasis and sensorimotor gating because the MeA has connections with the hypothalamus and basal ganglia [20,39]. In addition to the morphological alterations in the BLA after stress exposure, chronic stress causes neuronal hyperexcitability that facilitates fear learning [37,38]. In particular, the BLA has been examined because it has numerous executive connections with other brain regions including the prefrontal cortex, hippocampus, caudate nucleus, nucleus accumbens, and other amygdaloid nuclei [24,40]. Such changes occurring in the amygdala following stress exposure are different compared with changes in other brain regions. The most important difference is the duration of change. In other words, the morphological changes in the amygdala can persist for quite a long period after cessation of stressful situations, but morphological changes in the hippocampus and medial prefrontal cortex are reversible over a short period of time [24]. Furthermore, the persistent morphological changes in the BLA can be induced by an acute corticosterone treatment [34]. Changes in the amygdala frequently occur in concert with functional changes in other brain regions [24,40]. In addition, the responsiveness of each nucleus in the amygdala is heterogeneous [35]. In the present study, the common nucleus showing altered protein expression was the central amygdala (CeA) (Figures 4,5 and 6), which has been investigated to a lesser extent than the MeA or BLA. However, the CeA is responsible for directing the behavioral, autonomic, and endocrine responses in an anxious state [41,42]. Therefore, specific roles of newly identified genes in relation to neural plasticity of the CeA during the stress response deserve further study, and it will also be necessary to investigate which regions will show changes in functional connections with the CeA.

A single situation may not result in the same response in different individuals, and this is true in both humans and animals. Some people are susceptible to PTSD, whereas others are not, even though they experienced the same severe disaster [22,23]. We occasionally found such a phenomenon in experimental animals that were subjected to CIS. To produce the CIS-induced depressed animal model, we purchased inbred mice of the same age and treated them with identical conditions. Moreover, behavioral assessments of all mice after the CIS regimen were simultaneously conducted with the same protocol. Although mice experienced the same CIS regimen, their behavioral consequence had a somewhat wider range compared with the CTL mice (Table 3). In this study, we selected mice for immunohistological analyses of four candidates from the STR and CTL groups using their immobility duration and found that protein expression of candidates was significantly decreased in the STR-HI mice but not in the STR-LI mice (Figures 3,4 and 5). Because immobility in the FST is considered a representative depression behavior [43], the difference in the immobility duration in the STR group can be interpreted as individual stress sensitivity. The mice used in this study had the same genetic background. Thus, individual stress sensitivity may be an acquired trait, and we hypothesize that individual stress vulnerability is closely correlated with the stress sensitivity of one’s amygdala. Furthermore, the four genes reported in our current study may, at least in part, play a role in emotional behavioral changes related to stress vulnerability.

## Conclusions

In summary, we identified eight genes as stress responders in the amygdala using a CIS-induced depression model and confirmed that decreased protein expression of the products of four genes was found in the STR-HI mice. Thus, these four genes may be involved in the chronic stress-induced depressive behaviors and acquired stress vulnerability of the amygdala. Therefore, further study is necessary to investigate the neural connections that originate in the CeA where gene expression levels decreased, because communication between the amygdala and other brain regions is indispensible for the exact function of the amygdala. Moreover, evaluation of the effect of gene expression changes on neural plasticity can help us understand the meaning of gene expression changes in the amygdala of CIS-induced depressed mice.

## Abbreviations

BLA: Basolateral nucleus of the amygdala; CeA: Central nucleus of the amygdala; CIS: Chronic immobilization stress; CTL: Control group; CTL-MI: Control-mean immobility; DC: Decapitation; ECM: Extracellular matrix; FST: Forced swim test; Gdf10: Growth differentiation factor 10; Igf2: Insulin-like growth factor II; Igfbp2: Insulin-like growth factor binding protein 2; IHC: Immunohistochemistry; MeA: Medial nucleus of the amygdala; Pcolce: Procollagen C-proteinase enhancer protein; PTSD: Post-traumatic stress disorder; Q-PCR: Quantitative PCR; SPT: Sucrose preference test; STR: Stressed group; STR-HI: Stressed-higher immobility; STR-LI: Stressed-lower immobility.

## Authors’ contributions

SJ carried out CIS, behavioral assessments, and made the outline of the draft. YL set up the protocol for SPT and participated in behavioral assessments. GK created a protocol for IHC and participated in the IHC process. HS participated in the CIS procedure and assisted in animal care. DHL carried out statistical analyses. GSR participated in the IHC procedure and helped draft the manuscript. SSK participated in the design of the study and analysis of microarray data. GJC participated in the process of IHC and analysis of the results. WSC participated in the design of the study and helped draft the manuscript. HJK designed the study and wrote the manuscript. All authors read and approved the final manuscript.
